# Treatment costs of psoriasis in a tertiary-level clinic

**DOI:** 10.1186/1472-6963-14-344

**Published:** 2014-08-15

**Authors:** Anssi Mustonen, Mauri Leino, Kalle Mattila, Leena Koulu, Risto Tuominen

**Affiliations:** Department of Dermatology, Turku University Hospital and University of Turku, Turku, Finland; Department of Public Health, University of Turku and Primary Health Care Unit, Hospital District of Southwest Finland, Turku, Finland; University of Turku, Lemminkäisenkatu 1, 20014 Turku, Finland

**Keywords:** Psoriasis, Psoriatic arthritis, Cost, Phototherapy

## Abstract

**Background:**

The costs of psoriasis to a tertiary-level clinic vary considerably depending on the country of study and methods used. Hospitalisation and phototherapy have been significant cost components. This study was performed to estimate the distribution and relative magnitude of the costs of psoriasis to a tertiary-level clinic.

**Methods:**

Based on 233 patients, outpatient and phototherapy visits and the days hospitalised were collected from the treatment provider’s records. The visit costs represented true costs, used to charge the final payers. Patients were analysed according to their treatment modalities.

**Results:**

On average, hospitalised patients (3.4%) had 31-fold higher total costs than non-hospitalised patients (p < 0.0001). The costs of hospitalisations formed 45% of all the treatment costs to the entire study population. Phototherapy accumulated 19% of the overall treatment costs. Patients receiving biological drugs or both phototherapy and traditional systemic therapy had the highest costs of treatment.

**Conclusions:**

The current study indicates that a small percentage of all psoriasis patients generate a large proportion of the overall costs to a tertiary-level hospital. Treatment modality has a significant effect on the costs to a tertiary-level hospital.

**Electronic supplementary material:**

The online version of this article (doi:10.1186/1472-6963-14-344) contains supplementary material, which is available to authorized users.

## Background

Psoriasis is a lifelong disease with no known curative treatments. In several studies from different countries, the prevalence of psoriasis was estimated at 1.5%-2.8% of women and 2.3%-s2.9% of men [1, 2]. Around 10%-30% of patients with psoriasis also have psoriatic arthritis [3, 4]. A recent review considered only seven studies dealing with the costs of psoriasis [5]. Psoriasis is is thought to be one of the most costly dermatological diseases due to its high prevalence [6]. Estimates of per-patient costs vary considerably depending on disease severity [7, 8]. Treatment type and treatment failure in psoriasis treatments have a significant effect on the total costs of psoriasis treatment [9–11]. Patients with psoriasis are at increased risk of other serious illnesses, including cardiovascular disease, depression and increased risk of mortality, increasing the costs related to psoriasis [12–14].

The costs of psoriasis to a tertiary-level clinic vary up to 20 fold due to the different methods used [5, 15–20]. Hospitalisation is a significant cost component in many psoriasis cost-of-illness studies [5]. In general hospitalisation is a decreasing trend in psoriasis care [11, 21–23]. The proportions between outpatient and hospitalisation costs vary greatly between different studies. It seems that treatment patterns in different countries have a significant effect on the distribution of costs. There is only one previous study from Scandinavian social security systems concerning treatment costs of psoriasis and it was limited to a one-month follow-up.

Most of the psoriasis cost-of-illness studies have been conducted in patients with moderate- to-severe psoriasis though there is up to a fourfold variability in the Psoriasis Area Severity Index (PASI) [16–18]. The aim of this study was to estimate the distribution of costs and the cost of psoriasis treatment in a tertiary-level clinic.

## Methods

### Patient sample

The sample was based on psoriasis patients who had visited the dermatology clinic in Turku University Hospital (TUH) between 1 October 2009 and 30 September 2010 and were diagnosed as having psoriasis (Ps) or psoriatic arthritis (PsA). In the Finnish healthcare system, patients with mild psoriasis are usually treated at primary health care settings and only moderate-to-severe cases are referred to a tertiary-level clinic for further treatment. PsA patients visit the dermatology clinic because of skin symptoms and receive only dermatological treatment from the dermatology clinic; rheumatologists or internists in other clinics treat possible joint problems. Of the 498 patients attending the clinic during the study period, 428 had psoriasis and 70 had psoriatic arthritis. The patients were sent a questionnaire by mail; the mailing was sent again to those who did not respond initially. A total of 262 patients completed the questionnaire (52.6% of the total study sample). Patient selection is described in more detail by Mattila et al. [24].

### Ethical consideration

The ethical committee of The Hospital District of Southwest Finland approved the study. The patients received a written description of the sampling procedure and study purpose, as well as the planned use and storage of the information they were to provide. This was followed by a description of the subject’s rights according to the Helsinki declaration. The patients were asked to give written consent to use their medical records for the study.

### Clinical data

Of the 262 subjects, 29 patients did not give written consent for the right to use their medical records. Clinical information was collected from the medical records of 233 subjects who gave consent, for the same time period that was covered in the questionnaire data. Outpatient and phototherapy visits and the days hospitalised were collected from the treatment provider’s records. Different types of outpatient visits and hospitalisation days have varying costs. There were 12 types of outpatient visits. Each cost item is based on true costs, which are used to charge the local communities that finally cover the costs of their residents (Additional file 1). The costs include all medications, medical equipment, time used by doctors and other medical staff members and other expenses of the tertiary-level clinic, during outpatient visits, phototherapy or hospitalisation. During outpatient visits medications are usually not provided. However, infusible biologic medications (infliximab) can be administered during an outpatient visit. Other biologicals and drugs are purchased by patients from general pharmacies without any costs to the clinic, and thus, their costs were not included in this study. The costs for each visit type and hospitalisation were obtained from the hospital administration (Additional file 1). The actual costs per patient of laboratory and pathology tests were separately collected from the records as they are separately charged from the local communities. The data consisted of every test ordered by the dermatology clinic during the study period. A phototherapy visit had two different cost categories depending on the given phototherapy type (bath-PUVA, UVB). The costs were applied to each visit category collected from the patient records and were used for all cost computations.

The Finnish Social Insurance Institute (FSII) provided information on the medications used by patients. Patients were divided into subgroups according to their treatment modalities, as following: 1) only topical treatment (including vitamin-D-analogues, corticosteroids, creams and combinations of these), 2) phototherapy (UVB or PUVA), 3) traditional systemic medications (acitretin, cyclosporin, and methotrexate), 4) combination treatment (treatments from both groups 2 and 3 and 5) biological medications (adalimumab, etanercept, infliximab, and ustekinumab). Patients in groups 2–5 may have also received topical treatments and patients in group 5 may have also received phototherapy and/or traditional systemic medications. A patient could only be in one group as described above.

The PASI score and the diagnosis International Classification of Diseases (ICD-10) code of Ps (L40.0) or PsA (L40.5) were extracted from clinical records to classify the severity and the type of psoriasis. If there were many PASI values recorded from the same patient, the mean value for the study period was calculated and used in the analysis. Patients were also divided into subgroups for further assessment by diagnosis of psoriasis (psoriasis and psoriatic arthritis) and the severity of psoriasis.

### Statistical analyses

The statistical evaluation of the data was based on a chi-square test for proportions and a t test for means. The Pearson coefficients of correlation were used to examine the degree of relationship between two continuous variables. Linear regression models were used to study how different background factors affected the variation in treatment cost estimates. The distribution of the overall treatment costs were skewed so the data were converted to close to a normal distribution by natural logarithmic transformation and used as a dependent variable. In the analysis of the subgroup with PASI values, all patients without recorded PASI values were excluded. When analysing the subgroups with recorded PASI values (72 patients), patients were divided by the median value to more severe (PASI more than 5.5 [n = 37] and less severe psoriasis [PASI less or equal than 5.5, n = 35]). Only a few patients had PASI values above 10; using such a high index value for categorising would have produced a subgroup of severe Ps patients, which is too small for statistical analysis.

## Results

There were only minor differences in the patient characteristics of different treatment modality groups (Table 1). The annual cost of tertiary-level treatment per patient with psoriasis varied widely, from €32 to €43 842, with a mean of €1419 (Table 2). Half of the patients had costs less than €600 and 95% of patients had costs less than €2500. Patients with more severe psoriasis had on average significantly (p < 0.05) higher treatment costs (€2683) than patients with less severe psoriasis (€562). Patients receiving combination therapy or biological medications had the highest total costs (Table 2). Only one patient received an infusible biologic medication (infliximab) during outpatient visits. Patients with psoriatic arthritis had slightly but non-significantly lower total costs than patients with psoriasis.

**Table 1:** **Patient characteristics according to treatment modalities and to all patients (all treatment modalities were compared with topical therapy group in statistical comparisons with the t-test and chi-square test for proportions)**

	Topical therapy (n = 75)	Phototherapy (n = 63)	Traditional systemic therapy (n = 66)	Combination therapy (n = 17)	Biological therapy (n = 12)	All patients (n = 233)
Male	49%	57%	55%	65%	58%	55%
Mean age	56.8	52.9	63.3*	57.7	52.5	57.4
Disease duration	17.4	20.6	22.3	29.6*	21.5	20.8
Proportion of PsA patients	9%	25%	12%	12%	0%	14%
Mean PASI (n = 71)	5.8	7.3	5.4	8.1	7.1	6.5

*=p < 0.05, otherwise non-significant.

**Table 2:** **Annual costs (€) of psoriasis treatments per cost items for each treatment modality and to all patients (all treatment modalities were compared with the topical therapy group in statistical comparisons with the t test)**

	Topical therapy (n = 75)	Phototherapy (n = 63)	Traditional systemic therapy (n = 66)	Combination therapy (n = 17)	Biological therapy (n = 12)	All patients (n = 233)
Phototherapy costs	0	755.5^§^	0	764.6^§^	112.9§	265.9
Other outpatient costs^a^	340.7	382.4	453.0	444.1	4350.1**	468.8
Pathology costs	16.0	11.4	19.1	18.0	36.0	66.2
Laboratory costs	24.9	19.1	102.1***	71.7***	62.4*	
Hospitalisation costs	177.7	211.6	848.3	2509.2	4611.8*	617.9
Total costs	559.4	1381.3**	1422.4	3807.5**	6914.6***	1419.0

^a^ = Includes all outpatient visit costs, except those of phototherapy visits.

^§^ = Not applicable * = p < 0.05, ** = p < 0.01, *** = p < 0.001, otherwise non-significant.

A great majority of patients had only outpatient visits to the clinic. Only eight patients (3.4%) were hospitalised because of psoriasis during the study period, for an average of 7 days. However, the costs of these hospitalisations formed 45% of all the treatment costs in the entire study population. On average, hospitalised patients had costs that were 31-fold higher than non-hospitalised patients (p < 0.0001). Patients from all different treatment modality groups had been hospitalised; however, patients receiving combination therapy or biological medications had higher costs of hospitalisation (Table 2). All the hospitalised patients were in the more severe (PASI > 5.5) psoriasis group (p < 0.01). The hospitalised patients also had more outpatient visits but they received less phototherapy than non-hospitalised patients. Females were more likely to be hospitalised (p < 0.05).

Approximately a third of the patients received phototherapy and on average they received 14.8 phototherapy sessions per year. Phototherapy accounted for 20% of the overall treatment costs. The patients who received phototherapy had higher total costs than those who were treated with topical treatments only, but lower costs than patients in the other treatment modality groups (Table 2).

In a linear regression model when the effects of other factors were simultaneously controlled, being hospitalised and receiving phototherapy were the strongest predictors of higher treatment costs. Increasing age and income level were also significantly related to increasing costs. Sex and type of psoriasis (Ps or PsA) had a minor and non-significant effect on overall treatment costs (Table 3).

**Table 3:** **Linear regression model for studying the effects of the background factors on total costs**

Background factor	Beta-value	p<
Male	-0.054	0.591
Age	0.016	0.000
Disease duration	0.000	0.893
Income level	0.023	0.026
Diagnosis (Ps/PsA)	0.123	0.387
Hospitalisation	3.469	0.000
Phototherapy	1.223	0.000
R^2^	0.603	

ln(1+ total costs) as dependent variable.

## Discussion

Overall, the treatment costs of psoriasis are mainly generated by only a small proportion of patients who have more severe disease and particularly those that have been hospitalised. The more severe cases who are hospitalised are also at higher risk of treatment failure, and failure has been shown to be a strong predictor of increased costs [9, 10]. Consistent with our findings, in previous studies by Finzi et al. and Steinke et al., [11, 25] the great majority of the overall costs were generated by a small proportion of patients. Although hospitalisation has been a decreasing trend in psoriasis care [21–23], every effort to reduce hospitalisation days further would mean significant cost reductions [2]. In previous studies, hospitalisation has been estimated to be a more effective way of treating psoriasis than outpatient treatment [26]. In a more recent study, Steinke et al. [11] suggested that hospitalised patients require extensive treatments after leaving the hospital and reported a short time to relapse after hospitalisation, thus questioning the effectiveness of hospitalisation.

The proportion of hospitalisation of the total costs has varied considerably in previous studies [11, 15–19]. In the study by Sohn et al. [19], the cost for hospitalisation was almost fourfold greater than that estimated for hospitalisation in our study. In their study, hospitalisation represented more than 80% of the total costs to a hospital, which was almost twice the proportion in the current study. Conversely, in a Swedish study [17], the hospitalisation costs were only 3% of the total costs to the hospital. The differences in the magnitudes and proportions of hospitalisation of the total treatment costs could be due to patient selection. In some studies [15, 16, 19], the patient samples have included mainly or only subjects with very severe psoriasis. Patients with more severe disease are more likely to be hospitalised and more likely to receive more intensive, longer lasting and costly treatments, which was also observed in the present study.

When assessing the total costs of psoriasis, the cost estimates per visit or per day have a significant effect on the final cost. Our study was based on the actual cost used to charge the local communities. The diagnostic-related group unit costs per hospitalised day in this study were higher than the ones used in earlier studies [11, 16, 17, 19]. This increased the proportion of hospitalisation costs in the current study compared with other studies.

The sample of patients in the present study could be expected to represent typical tertiary-level patients in Finland as all patients who had visited the TUH dermatology clinic with a psoriasis diagnosis during the period of one year were included without any selection. Thus, the whole variety of psoriasis cases was included. In Ghatnekar et al. [17], the costs were based on observations from a period of one month—November-December, the time when psoriasis is expected to flare in Scandinavian countries. A follow-up period of one month may also be too short to catch various patient cases, which may be the reason why their sample included only one patient who had been hospitalised. Longer follow-up time could be expected to produce more stable cost estimates and decrease the possible effect of the fluctuating nature of psoriasis.

In Ghatnekar et al. [17], one-fifth of the costs were from biologic medication whereas in our study only one person received biologic medication during outpatient visits. It is not clear how big a proportion of the patients in the Swedish study received biologics during treatment visits and how many had them administered at home; thus a direct comparison of the findings is not possible. Because only biologics that were infused in the hospital during an outpatient visit or while the patient was hospitalised were considered, the outpatient costs for the biologic treatment were high, because the cost of infliximab was included in the visit cost. In our recently published study [27], the costs of biologic medications were significant and comprised 45% of the total costs of medications. For the patients receiving them, an annual average cost of €15 000 was estimated. In Finland, biologics administered by patients themselves are reimbursed by the FSII and thus do not comprise costs to a tertiary-level clinic. This kind of reimbursement system may have led to reduced use of infusible biologics and favoured the self-administrable biologics that have no costs to the dermatology clinic.

The introduction of biologics has been shown to increase the costs of medication, but to reduce the other treatment costs especially in high-need patients [28]. In our study the hospitalisation rates and overall treatment costs to a tertiary-level clinic were not significantly lower for patients receiving biologic medications. This may be due to using biologic medications only in patients with already high overall costs, and the high outpatient costs may have been partly due to the patient receiving infliximab in an outpatient setting. Our cross-sectional study design did not allow for further analysis on the impact of initiating biologic medications, which may have added to the overall treatment costs.

In studies [15, 18] where the PASI-values are very high, only the most severe psoriasis patients have been selected and this might have an effect on the cost estimates. Psoriasis has a tendency to flare and fluctuate so studies of patients with very high PASI values may overestimate the total costs as patients with a current flare need more treatments than patients who are in remission. Longer follow-up time decreases the effect of the fluctuation on the costs for care. In a recent study by Steinke et al., the PASI values were collected in the same manner as in our study with the exception of the use of maximum PASI found from the records during the study period, whereas in this study all values from the study period were considered and an arithmetic mean was used as a variable [11]. Only a proportion of patient records in this study included PASI values. There is no evidence of any selection of patients with or without PASI recordings, but due to the proportion of missing PASI values these sub-analyses should be considered with more caution.

Phototherapy may be a significant cost burden. However, the cost estimates of phototherapy in different studies have varied greatly [15–17]. Our findings were close to those of the Swedish study [17] in which almost a third of the costs were from phototherapy. In some studies, phototherapy has formed less than a tenth of the treatment costs [15, 16]. Large differences in phototherapy costs may be due to unit costs used in different studies. Phototherapy is time consuming and requires other resources from the provider. In the present study, all unit costs were based on actual costs, obtained from the registers, which are also used to charge the final payers. Thus, direct comparison of the present findings with previous studies [15, 16] which may have used rough cost approximations, may be problematic.

The present study data was based on actual numbers of various types of visits for the period of one year collected from the records, which could be expected to produce reliable estimates. In comparison, in Ghatnekar et al. [17], the numbers of outpatient visits for one year were extrapolated from data for one month, and the numbers of outpatient visits obtained from hospital records were four times the numbers estimated by the patients in the study.

There are no secondary-level hospitals with dermatology clinics in the administrative region where the current study was performed. If available, patients with moderate-level psoriasis would probably have been at least partly treated in secondary-level clinics. Consequently, the sample may have more patients with moderate psoriasis than many other studies with a tertiary-level setting, which may affect also the average cost estimates. Ghatnekar et al. [17] has shown that treatment costs for an average patient with psoriasis in secondary-level hospitals were almost half of those in tertiary-level hospitals. The disease severity of our patients, based on PASI values, was approximately the same as the overall severity of the patients in secondary and tertiary-level hospitals [17], indicating that the present study patients represent the whole variety of moderate to severe psoriasis cases in the hospital district, although the sample was derived from the records of a tertiary-level clinic.

## Conclusions

In previous studies, the cost estimates for psoriasis varied greatly between studies conducted in different societies. There is a need to harmonize the study field to be able to compare the results of different studies [5]. Decreasing hospitalisation may result in a significant cost reduction. The current study indicates that a small percentage of all psoriasis patients generate a large proportion of the overall costs.

## Electronic supplementary material

Additional file 1: **Price list.** Hospital District of Southwest Finland 2010 (translated from Finnish original) Dermatological unit. (DOCX 14 KB)
