# Supplementary material for: Treatment costs of psoriasis in a tertiary-level clinic
Source: BMC Health Serv Res. 2014 Aug 15;14:344. doi: 10.1186/1472-6963-14-344 (PMC4141106; doi:10.1186/1472-6963-14-344)
Supplement: Supplementary file 1 — Additional file 1: Price list. Hospital District of Southwest Finland 2010 (translated from Finnish original) Dermatological unit. (DOCX 14 KB) [file 12913_2014_3450_MOESM1_ESM.docx]

Price list: Hospital District of Southwest Finland 2010 (translated from Finnish original)

Dermatological unit

This list is used to charge the local communities, which cover the costs of patients’ treatments.

| Visit type | Description | Cost (euro) |
| --- | --- | --- |
| P1 | Serial treatment (under 30 min), e.g. UVB treatment | 32 |
| P2 | Short consultation (doctor), calling the patient, skin care by a nurse (30 – 60 minutes) | 51 |
| P3 | Outpatient visit (doctors office, no procedures), bath-PUVA, skin care by nurse (over 60 minutes) | 100 |
| P4 | Demanding outpatient visit (doctors office, includes possible procedures, e.g. skin biopsy) | 167 |
| P5 | Demanding outpatient visit to a specialist | 266 |
| P6 | Outpatient visit demanding an extensive operation, or extensive planning for rehabilitation | 534 |
| P7 | Outpatient visit demanding multiple operations, day-care in a dermatological ward | 1 129 |
| P8 | Outpatient visit demanding an expensive medication (e.g. infusion of a biological drug) (includes the cost of the drug) | 2 222 |
| DRG 272 | Inpatient treatment of a difficult skin disorder | 2 666 |
